# Effect of Mixing Time on Properties of Whole Wheat Flour-Based Cookie Doughs and Cookies

**DOI:** 10.3390/foods12050941

**Published:** 2023-02-22

**Authors:** Somali Dhal, Arfat Anis, Hamid M Shaikh, Abdullah Alhamidi, Kunal Pal

**Affiliations:** 1 Department of Biotechnology and Medical Engineering, National Institute of Technology Rourkela, Rourkela 769008, India; 2 SABIC Polymer Research Center (SPRC), Department of Chemical Engineering, King Saud University, P.O. Box 800, Riyadh 11421, Saudi Arabia

**Keywords:** cookie, dough, fracturability, mixing, spreadability, texture, whole wheat flour

## Abstract

This study investigated if whole wheat flour-based cookie dough’s physical properties were affected by mixing time (1 to 10 min). The cookie dough quality was assessed using texture (spreadability and stress relaxation), moisture content, and impedance analysis. The distributed components were better organized in dough mixed for 3 min when compared with the other times. The segmentation analysis of the dough micrographs suggested that higher mixing time resulted in the formation of water agglomeration. The infrared spectrum of the samples was analyzed based on the water populations, amide I region, and starch crystallinity. The analysis of the amide I region (1700–1600 cm^−1^) suggested that β-turns and β-sheets were the dominating protein secondary structures in the dough matrix. Conversely, most samples’ secondary structures (α-helices and random coil) were negligible or absent. MT3 dough exhibited the lowest impedance in the impedance tests. Test baking of the cookies from doughs mixed at different times was performed. There was no discernible change in appearance due to the change in the mixing time. Surface cracking was noticeable on all cookies, a trait often associated with cookies made with wheat flour that contributed to the impression of an uneven surface. There was not much variation in cookie size attributes. Cookies ranged in moisture content from 11 to 13.5%. MT5 (mixing time of 5 min) cookies demonstrated the strongest hydrogen bonding. Overall, it was observed that the cookies hardened as mixing time rose. The texture attributes of the MT5 cookies were more reproducible than the other cookie samples. In summary, it can be concluded that the whole wheat flour cookies prepared with a creaming time and mixing time of 5 min each resulted in good quality cookies. Therefore, this study evaluated the effect of mixing time on the physical and structural properties of the dough and, eventually, its impact on the baked product.

## 1. Introduction

Cookies are delicious, healthy, and convenient baked delicacies. They have a long shelf life and may be obtained with little effort. Countless varieties of cookies exist, each with its own unique flavor and texture, thanks to the wide range of ingredients that may go into the dough. Most cookie dough recipes call for wheat flour, sugar, oil, and water. The reason for this is that these ingredients provide the maximum taste. Ingredients such as salt, eggs, and baking powder are only icing on the cake. The dough and end product (cookie) quality are determined by the processing parameters and the composition, amount, and ratio of the specified ingredients [1]. Wheat flour is an essential component of cookie dough. The presence of gluten proteins in wheat flour is the primary reason for the dough’s ability to retain its elasticity throughout the baking process [2]. The cookies benefit from gluten in terms of their organoleptic qualities; nevertheless, an excessive quantity of gluten might cause the baked product to be tough and chewy [3]. Fat (often butter) is typically added in cookie recipes to prevent these issues from occurring, since it slows down the formation of the gluten network by coating the protein strands and reducing the strand development. This process shortens the gluten strand in cookies and makes them more tender and crumbly [4]. Butter also provides flavor to the cookies, which is why it is always favored over margarine or vegetable shortenings. Aside from flavoring the cookies, butter also contributes to their form and structure. Sugar, another vital ingredient, contributes to the sweetness and influences the shape and texture of the cookies. When butter and sugar are combined, the resulting buttercream is considered to absorb air into the fat, resulting in a fluffy texture for the baked cookies. Therefore, sugar functions as a softening/tenderizing agent to regulate macromolecular transformations and contributes to the system’s emulsive–colloidal stability. Finally, the inclusion of water aids in developing and softening dough [5].

The quality of cookies is profoundly affected by processing variables such as dough preparation method, creaming time, mixing time, cookie thickness, baking time, and temperature. There are different methods for cookie dough preparation, i.e., all-in-dough preparation and creaming method [6]. The former method is a single-step method wherein all ingredients (both dry and wet ingredients) are mixed to produce a non-extensible dough. The latter method comprises two steps. The first step is the “cream-up” step, where sugar and fat are mixed. In the second step, called the “dough-up” step, the rest of the ingredients are mixed with minimal mixing to form a non-extensible and non-elastic dough containing minimum gluten. When butter and sugar are creamed together, they produce a homogeneous, frothy, and creamy mixture. This step forms a base to which the remaining dry ingredients are added. Additionally, it helps to aerate the cookie dough by producing minute air pockets that, after the cookies have been cooked in the oven, will cause the cookies to puff out. Without the creaming step, the cookies risk becoming thick and flat, adversely affecting their organoleptic features. While processing the cookie dough, the dough is refrigerated for an hour after mixing. This period is often termed the “standing period”. During this time, the water is passively absorbed by the starch and protein present in the flour. As a result of standing after mixing, cookie dough becomes firmer and less sticky. Hence, the “standing period” has been reported to stabilize and decrease quality discrepancies when the dough is used for baking purposes [6].

Similarly, the dough mixing time also represents an important parameter that decides the textural properties of the final baked product. Researchers have reported that mixing time affects the cookie hardness, which has been attributed to the fact that dough rheological properties change during the mixing process [7,8]. The process of depolymerization of the glutenin macropolymer (GMP) that occurs during dough kneading is significantly connected to the alterations mentioned above [9]. Long mixing times decrease GMP’s molecular weight, and glutenin polymers are released from the dough by being ruptured. Dough with poor consistency and a rough texture is the consequence of glutenin’s partial depolymerization, which creates molecules with a low molecular mass. Many other scientists have seen similar phenomena. According to Mancebo et al. [10], wheat proteins regulate particular dough qualities and the quality of processed food items, such as the spread factor [10]. Different types of wheat flour, such as whole wheat flour (WWF) and refined wheat flour (RWF), need different mixing times for making cookie dough. WWF contains the entire wheat kernel, whereas RWF is produced by removing the bran and germ layer of the wheat kernel. Whole wheat contains a high gluten concentration, thus improving the elasticity of flour [11]. The gluten in WWF makes it easier to roll out thin sheets, but doing so requires more attention to mixing time than RWF does. RWF produces gluten structures that are stronger than WWF’s, which is why cookies made with RWF are chewier than those made with WWF. Apart from the processing parameters, WWF and RWF also differ in their chemical constituents. WWF is nutritious and contains higher antioxidant properties due to ferulic acid, caffeic acid, Vitamin E, and phytic acid-like bioactive compounds [12]. On the other hand, RWF lacks these beneficial compounds and also imparts adverse health effects (e.g., hyperacidity, constipation, and obesity) in the long run [13]. Furthermore, RWF has a very high glycemic index, which means that it releases sugar into the bloodstream quickly. To combat this, the pancreas creates insulin spikes that continue for longer. Unfortunately, this leads to inflammation and insulin resistance, which ultimately results in type II diabetes.

According to the prior discussion, the kinds of wheat flour and the processing parameters are two important factors that significantly impact the textural characteristics of baked products. The method through which the dough is processed impacts the textural features of the finished product and the quality attributes of the dough itself. However, there has been no study on the impact of mixing time on WWF-based cookie dough or cookies. Hence, this study aims to investigate the effect that the time spent on mixing has on cookie dough made using WWF and baked cookies. Therefore, in this study, the characteristics of cookie dough under different mixing times were investigated using texture (spreadability), moisture content, and impedance analysis. Further, the baked cookies were evaluated using spread factor, surface topology, impedance, moisture content, and texture analysis (hardness and fracturability).

## 2. Materials and Methods

### 2.1. Raw Materials

Whole wheat flour (Aashirvaad, manufactured by ITC Limited in Kolkata, India), salted and pasteurized butter (produced by Amul Pvt. Ltd. Anand, India), baking powder (manufactured by Weikfield Food Pvt. Ltd. in Nalagarh, India), sugar, and salt (manufactured by Tata Chemicals Ltd. in Bombay House, Mumbai) were sourced from local grocery stores. The whole wheat flour had the following nutritional content per 100 g: 396 kcal energy, 77 g carbohydrate, 10.8 g protein, 5.6 g sugar, 11.1 g dietary fiber, and 1.8 g fat. The flour contained 10.5% moisture and 2.2% ash, and the particle size was <100 µm.

### 2.2. Preparation of Cookies

The cookies were prepared using the listed ingredients (Table 1) per the traditional creaming process. At the beginning of the process of making the dough, butter and powdered sugar were creamed together with an electric hand mixer (OHM-207, 150-Watt, Orpat) set to speed-1 for 5 min to achieve the consistency of mayonnaise. The butter and sugar were at room temperature (25 °C). Then, the other dry ingredients, which included flour, baking powder, and salt, were added and combined at speed-1 for different time periods (Table 1). After adding 25 g of water to the mixture described above, a dough with a crumbly consistency was produced. After cookie dough had been made, it was wrapped in a food wrapper, placed in the refrigerator, and allowed to chill (4 °C) for an hour [14]. Following chilling, the cookie dough was hand shaped into a thin flat sheet of uniform thickness. After that, the flat sheets were cut into circles (diameter: 50 mm; the weight of each dough piece: 15 g) using a cookie cutter. Twelve cookies were produced from each batch of cookie batter. After cutting the cookies, they were placed on a baking sheet lined onto the microwave pan and then cooked in a microwave oven (Samsung Smart Oven), operating in the convection mode, at 180 °C for 25 min. After removing the cookies from the oven, they were allowed to cool at room temperature for 2 h before being transferred to an airtight plastic container.

### 2.3. Impedance Analysis of Dough

The impedance was measured using the impedance analyzer (Digilent, analog discovery 2, National Instrument, Austin, TX, USA). In this study, a pair of stainless steel electrodes (diameter: 1 cm; distance: 1 cm) was inserted into the dough sample, and the impedance was measured in the frequency range of 1 Hz to 1 kHz [15].

### 2.4. FTIR

The IR-absorption spectra of the dough samples were obtained using an FTIR spectrophotometer (Alpha-E; Bruker, Bremen, Germany). The instrument was coupled with an attenuated total reflectance (ATR) Zinc selenide (ZnSe) crystal. The samples were scanned in the range of 4000–500 cm^−1^, each with 25 scans at a spectral resolution of 4 cm^−1^. The FTIR spectrum of cookies was recorded by using the powdered form of cookies and following a similar procedure to that of dough [16].

After eliminating the baseline, the FTIR spectrum was deconvoluted with Gaussian curves using the “Multiple Peak fit” tool and Levenberg–Marquardt algorithm featured in the Origin Pro (v9.1, Northampton, MA, USA) package. The numerical deconvolution was carried out to estimate the proportion of main bands (OH band, Amide I, and starch area) in the dough samples. R^2^ > 0.99 and χ^2^ < 0.001 were the parameters considered while performing the deconvolution. Bands attributed to a certain conformation were added together and divided by the overall area of the associated major band. This was then used to compute the percentage contribution of the various kinds of conformations to the total area of all components.

### 2.5. Physical Dimensions

A digital caliper was used to measure the thickness (T) and width (W) of the cookies after they had been cooked. To obtain the spread ratio of cookies, the diameter of cookie was divided by the thickness of the baked cookie [17,18].

### 2.6. Microscopic Analysis

#### 2.6.1. Dough Microstructure

The microstructure of the different dough samples was visualized under an upright bright-field compound microscope (Leica Microsystems, model: DM750, GmbH, Wetzlar, Germany), and the images were captured with an ICC 50-HD camera [19]. The segmentation analysis of the water regions was carried out using ImageJ software (NIH, Bethesda, MD, USA).

#### 2.6.2. Surface Topology of Cookies

A Stereo Zoom microscope (SM-2TZ, AMscope, Irvine, CA, USA) and an external eyepiece lens camera (MD500, AMscope, Irvine, CA, USA) were used to visualize the surface of the cookies at a magnification of 4×.

### 2.7. Moisture Content

The total moisture content of the dough and cookies was analyzed using digital moisture balance (LT-76, Dolphin Pvt. Ltd.). Accurately weighed (~2 g) crushed cookie samples were placed on an aluminum pan. The samples were then heated at 180 °C. The percentage of mass loss of the formulations during the drying process was noted. Measurement was performed in triplicate from each dough sample [20]. The percentage moisture content (%M) was determined using Equation (1).

(1) $$\%M=\frac{M_{t}-M_{0}}{M_{0}}\times 100$$

where %M is the percentage of moisture content, $M_{t}$ is the mass at the end of the drying process, and $M_{0}$ is the initial mass.

### 2.8. Texture Analysis

#### 2.8.1. Dough Spreadability

A texture analyzer (TA-HD plus apparatus, Stable Microsystems, Godalming, Surrey, UK) coupled with a 45° Perspex conical probe was used to test the spreadability of the dough. At room temperature, the texture analyzer was operated with a load cell of 50 N. The dough was loaded and flattened in the bottom Perspex cone. At a speed of 1 mm/s, the top Perspex cone was permitted to penetrate the dough sample. The penetration was set to end at a height of 2 mm from the bottom of the cone. The male cone then returned to its original position at the same pace. For each dough sample, the experiment was performed in triplicate [21].

#### 2.8.2. Stress Relaxation of Dough

The setup for the stress relaxation study was similar to that used in the spreadability study. The dough sample was placed into the lower Perspex cone and placed into the cone holder. The measurements were taken in compression mode, in which the conical probe penetrated the dough to a distance of 5 mm after a trigger force of 5 g. The pre-test, test, and post-test velocity was 1 mm/s, 0.5 mm/s, and 1 mm/s, respectively. This constant compressive strain was applied to the sample for 60 s. Each test was conducted at room temperature (25 °C) with three replications. Force was recorded continuously as a function of time t. The % S.R. of the dough was calculated using the force values in Equation (2) [22].

(2) $$\%SR=\;\frac{F_{0}-F_{60}}{F_{0}}\times 100$$

where $F_{0}\;$ is the maximum force, and $F_{60}$ is the residual force.

#### 2.8.3. Puncture Strength of Cookies

The fracturability of the cookies was determined with the help of the texture analyzer equipped with a 50 kg load cell Heavy Duty Platform. Cookies were placed on a platform and punctured to a distance of 1 mm after a trigger force of 5 g was achieved with a 3 mm cylindrical stainless steel probe (P/3). The pre-test, test, and post-test speed was 0.5, 0.5, and 1mm/s, respectively [23].

#### 2.8.4. Three-Point Bending Test of Cookies

The hardness of the cookies was measured by determining the texture analyzer (TA-HD plus apparatus, Stable Microsystems, Godalming, Surrey, UK). The texture analyzer was equipped with a 3-point bending rig (HDP/3PB) and a 50 kg load cell Heavy Duty Platform for the experiment. The cookie was placed on the adjustable support, placed 2 cm apart. Then, the compression was applied using a flexure attachment at a speed of 1 mm/s and a trigger force of 5 g until the cookie was broken into two halves. The cookie hardness was recorded as the maximum peak force required to break the cookie with the flexure attachment. The experiment was performed in triplicate for each cookie sample, and the average values were reported [24].

### 2.9. Statistical Analysis

Triplicate runs of the experiments were conducted, and the results were summarized as average ± standard deviation (SD). For the statistical analysis, we used IBM’s SPSS Statistics 20. (Inc., Chicago, IL, USA). Each parameter’s statistical significance was determined using a one-way analysis of variance (ANOVA) and Tukey post hoc test, with a significance level of *p* < 0.05 considered to indicate a statistically different value [25].

## 3. Results and Discussions

### 3.1. Analysis of Dough

#### 3.1.1. Visual Appearance of Dough

**Figure 1:**
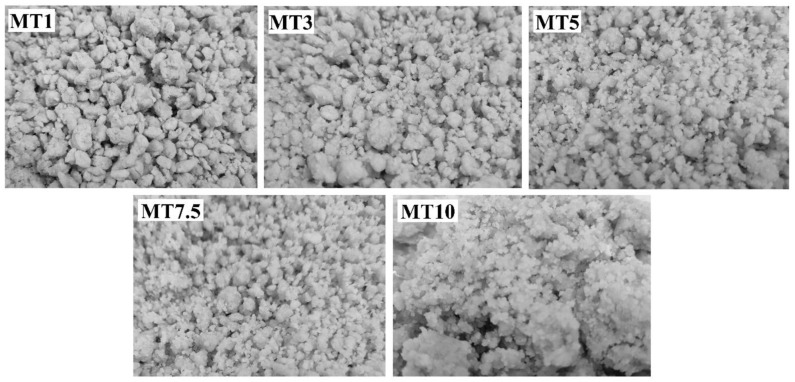
Visual appearance of the dough after mixing.

The primary purpose of mixing is to mechanically hydrate the flour particles so that they can create uniformly small and large crumbs for the dough (Figure 1). In MT1, larger dough particles were observed after mixing. This indicates that a mixing time of 1 min is insufficient to homogenize the dough mixture properly. An insufficiently mixed dough may have an uneven hydration of the wheat flour, resulting in a lack of gluten network development [26]. The dough particles were properly homogenized with an increase in the mixing time, resulting in small dough crumbs. However, when the mixing time exceeded 7.5 min, the dough became stickier and harder, thereby losing its crumbling property. It has been stated in the literature that excessive mixing can overdevelop gluten, break a dough’s well-developed gluten network, and raise the dough’s temperature to the point where the protein is denatured [27,28]. Therefore, an optimum mixing time was determined depending on the flour used. Studies have reported that the mixing time significantly affects the cookie hardness and spread factor [5]. Softer cookies with tender bites can often be achieved by combining a longer creaming time with a shorter mixing time.

#### 3.1.2. Moisture Content of Cookie Dough

**Figure 2:**
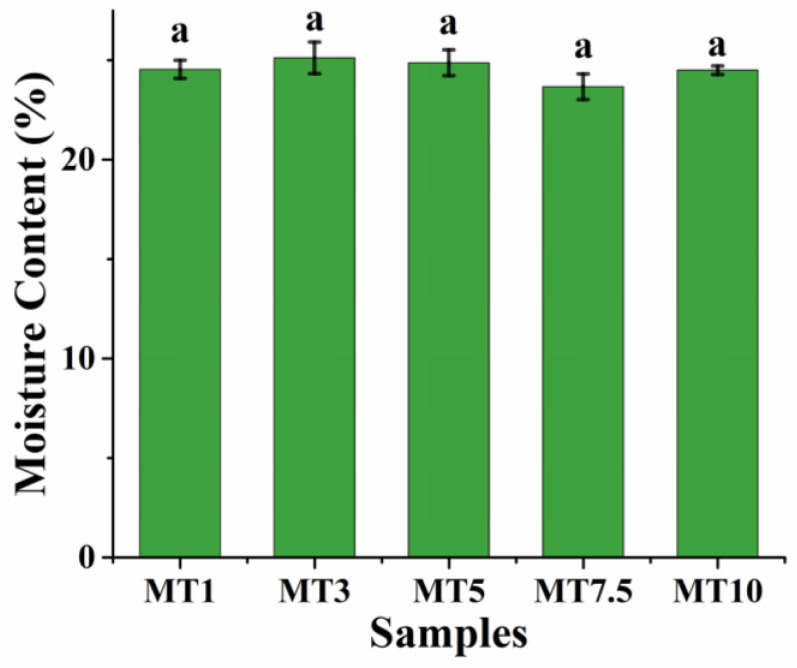
Moisture content profile of the cookie dough. Error bars represent the standard deviation of three replicates. For each chart, not sharing the same letter(s) are significantly different by Tukey HSD test.

The textural and rheological characteristics of flour-based products are determined by the amounts of starch, protein, and water used in their production [8,29,30]. Dough relies heavily on the interaction between flour and water; therefore, wheat flour is just one of the fundamental elements. When flour and water are mixed together, a network of protein and starch particles is formed, giving dough its elasticity and ductility [31]. The rheological characteristics of cookie dough may be drastically changed by even a 1% shift in moisture content [22,32]. High-quality end products depend on the moisture content of the WWF-water system. Previous studies have suggested that the dough’s preparation methods also affect the final properties of the dough and the product [31]. Quick and uniform mixing of the wheat, water, and other components is achieved throughout the mixing process, resulting in the flour’s hydration and the formation of a dough with high durability. Usually, cookie dough has a high percentage of water, approximately 20–25% of the total dough weight. The MT1 dough showed a moisture content of ~24.50%. A further increase in the dough mixing had an insignificant change in the moisture content of the dough samples (*p* > 0.05) (Figure 2). This result was expected as the dough’s composition was the same for all the samples.

#### 3.1.3. Dough Microstructure

**Figure 3:**
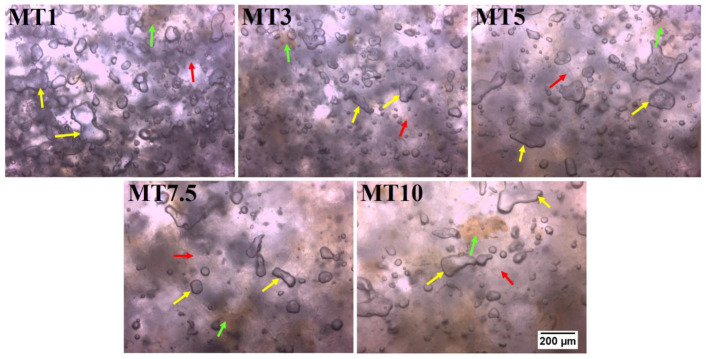
Bright-field micrograph of dough samples (Magnification 10×).

Figure 3 represents bright-field micrographs of the dough samples. The darker matrix is the starch matrix (shown by the red arrow) homogenously distributed throughout the sample. Some yellow-brown patches representing the bran particles were also observed in the micrographs (shown by the green arrow). The most interesting observation from the micrographs is the distribution of water molecules in the dough samples. MT1 showed many small and large water agglomerates (shown by the yellow arrow), depicting that the water molecules were not absorbed into the starch matrix. As the mixing duration increases, the water molecules get absorbed into the starch matrix, resulting in fewer water agglomerates in the samples (MT3-MT10). Interestingly, MT3 showed more homogenously distributed matrix and water molecules than the other samples. Compared to MT3, the rest of the dough micrographs showed coalescence of water molecules resulting in larger water droplets. One probable reason for this can be the absence of emulsifiers in the dough matrix. Emulsifiers stabilize the dough matrix by interacting with the fat and water molecules [33]. The absence of emulsifiers might have led to the destabilization of the emulsion system at higher mixing times.

**Figure 4:**
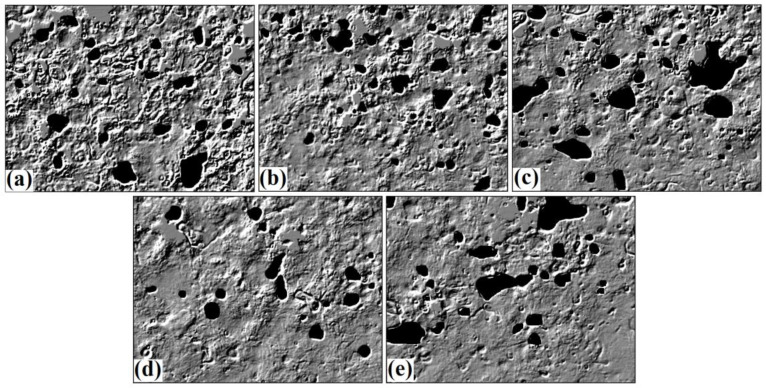
Segmented regions of water in the dough samples (**a**) MT1, (**b**) MT3, (**c**) MT5, (**d**) MT7.5, and (**e**) MT10.

We further analyzed the water regions through segmentation analysis (Figure 4). The percentage of the water regions in MT1, MT3, MT5, MT7.5, and MT10 were 6.48, 7.86, 11.47, 4.12, and 9.22%, respectively. Figure 4a suggests that MT1 had many water regions distributed throughout, which might indicate that all the water molecules were not absorbed into the dough matrix. MT3 showed that the percentage of water region slightly increased compared to MT1; however, much smaller regions were obtained in the case of MT3. MT5 showed larger water agglomerates with a high percentage of water regions. MT7.5 showed a lower portion of water regions than the rest of the samples. Lastly, the analysis of MT10 indicated the presence of larger water agglomerates (Figure 4e).

#### 3.1.4. FTIR Analysis of Dough

**Figure 5:**
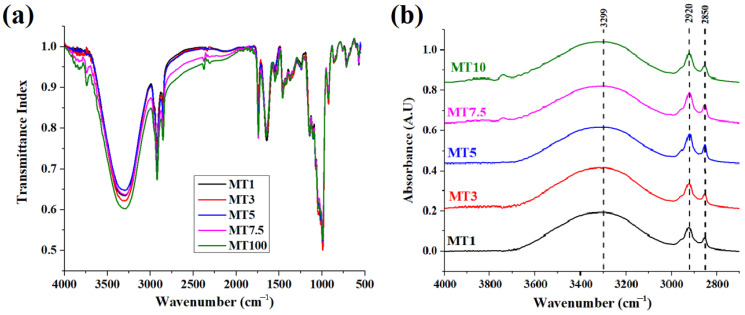
Graphs representing (**a**) FTIR spectrum of dough samples and (**b**) Absorbance spectrum of water region in the range of 4000–2800 cm^−1^.

FTIR spectroscopy was used to examine the variations in the dough and wheat gluten that resulted from varying mixing times to comprehend better the connection between macroscopic dough strength and aggregation behavior [34,35]. Figure 5a shows the typical ATR-FTIR spectra of the dough samples. The region in the range of 4000–1500 cm^−1^ is called the functional group region, and the region below 1500 cm^−1^ is called the diagnostic region.

**Figure 6:**
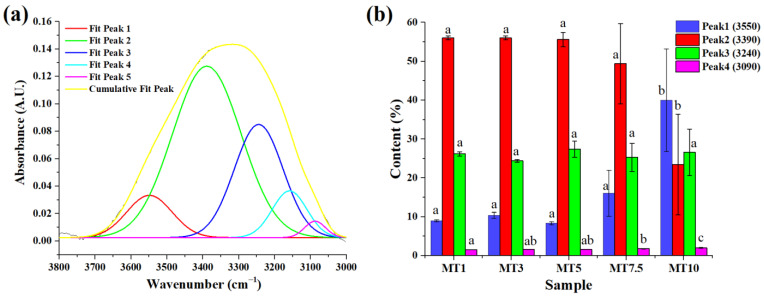
Graphs showing (**a**) a representative deconvoluted band of the water region and (**b**) variation of different peaks obtained in the water region concerning different samples. The alphabets above columns of the same color denote statistically significant (*p* < 0.05) differences.

All the samples showed broadband between 3800 and 2500 cm^−1^ (Figure 5b), which was composed of one broad peak at ~3300 cm^−1^ followed by two sharp peaks at 2920 and 2850 cm^−1^. The former broad peak can be attributed to the O-H stretching due to hydrogen bonding. This peak can be analyzed to study the changes in the water populations crucial for the gluten network formation. FTIR band analysis of water populations may indicate a reduction or rise in strong/weak hydrogen bonds between proteins and water molecules and a deficiency or excess of free water in the gluten network. Therefore, we carried out numerical deconvolution of the OH stretching vibration region (3800 and 3000 cm^−1^), resulting in five Gaussian peaks (as described by Walrafen et al. (1986) and Laurson et al. (2020)) to describe the changes in the water structure due to the difference in mixing times [36,37]. According to Walrafen, the OH band comprises the five distinct overlapping bands that are situated at 3090, 3220, 3393, 3540, and 3625 cm^−1^. The peaks obtained in the high wavenumber were correlated to the molecules involved only in water–water interactions. Conversely, vibrations obtained at the lower wavenumber were usually attributed to the molecules forming strong H-bonding [38]. An extra peak (~3158 cm^−1^) was also considered in this study to obtain high convergence during peak fitting (Figure 6a). The numerical deconvolution of the samples resulted in four peaks in each sample; however, no peak was obtained at 3625 cm^−1^ [39] (Figure 6b). For all the peaks obtained during the deconvolution, a shift of± 10–20 cm^−1^ was observed for all the peaks. Such shifting can be explained due to the difference in the composition of dough compared to the results obtained by Garcia-Valle et al. (2021) [39]. Peak1 corresponded to the symmetric vibrations of the –OH group associated with the non-hydrogen bonded or free water molecules. MT1–MT7.5 showed statistically similar values for Peak1 (*p* > 0.05); however, the value increased significantly at MT10 (*p* < 0.05). This means a higher mixing time might not support hydrogen bonding among the water molecules. Peak2 was dominant in all the dough samples (>50%) except MT10, where it decreased significantly to 25%. The higher concentration of Peak2 in most of the samples implies the presence of a significant number of water molecules with weak hydrogen bonding between them. The other two peaks, Peak3 and Peak4, are associated with the vibrations resulting from the strongly bonded water molecules through hydrogen bonding. The percentage of Peak3 was similar for all the samples, suggesting that a change in the mixing time did not affect this peak. On the contrary, Peak4 showed variations with changes in the mixing time. MT1, MT3, and MT5 had comparable Peak4 levels to MT7.5 and MT10, which had much higher content. (*p* < 0.05). According to the literature, peaks obtained at 3090 cm^−1^ or the region below it can be attributed to the Fermi resonance of the overtone of OH-in-plane bending with the OH vibration of strongly hydrogen-bonded water molecules. The presence of strongly bonded water molecules suggests that the proteins and fiber of WWF may subsidize to the stronger bonding of water molecules with the flour particles during dough processing.

**Figure 7:**
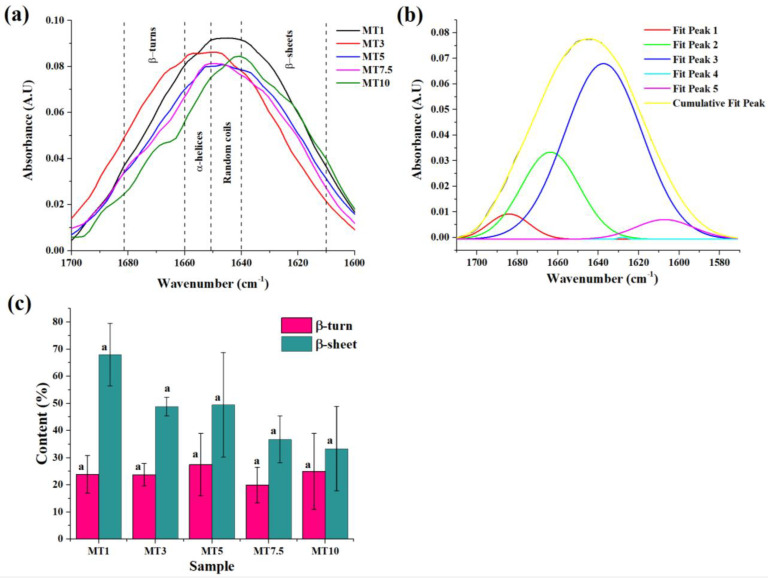
Graphs showing (**a**) Amide I region containing different protein secondary structures in different samples, (**b**) a representative graph of the deconvoluted Amide I region, and (**c**) variation of different structures obtained in the Amide I region with respect to different samples. The alphabets above columns of the same color denote statistically significant (*p* < 0.05) differences.

Further in the spectrum, C-H stretching modes were responsible for bands between 2800 and 3000 cm^−1^. The two sharp peaks may account for the asymmetric and symmetric stretching vibrations of the aliphatic CH_2_ groups at 2920 and 2850 cm^−1^ (Figure 5b). C=O stretching of the carbonyl group can account for the significant rise at 1742 cm^−1^. Following this sharp peak, a small peak was obtained at ~1640 cm cm^−1^. This peak might be associated with the C=O stretching of the Amide I group in combination with the O-H bending of water [34,35]. The gluten content of the dough greatly influences this phenomenon since a more significant number of gluten proteins is often associated with a greater water absorption capacity. Changes in the polymeric protein fraction during mixing determine cookie dough’s quality characteristics. Unlike traditional bread or noodle dough, which has a 35–60% moisture content, cookie dough is much drier (<30% moisture content) and is crumbly due to discontinuous gluten formation. The discontinuity in gluten network formation can be attributed to the presence of a shortening agent such as butter. Gaussian curve fitting was applied to the amide I region to emphasize the conformational changes in the secondary protein structure and to resolve the peaks in the 1700–1600 cm^−1^ region (Figure 7a). Figure 7b is a representative graph of the deconvoluted region. This region contains overlapping bands relating to the β-sheets (1610–1640 cm^−1^), random coil (1640–1652 cm^−1^), α-helices (1652–1660 cm^−1^), and β-turns (1660–1685 cm^−1^) [39]. When a sheer force (e.g., mixing) is applied, these protein molecules undergo conformational rearrangement to achieve a stable state with minimal energy. Reports have suggested that α-helices and β-sheets were relatively orderly and stable, while β-turns and random coils were disordered [40]. The increase in the mixing time led to a decrease in the intensity of the amide I band (Figure 7a), suggesting that a higher mixing time might not support the formation of a stable amide bond. The numerical deconvolution of the peaks suggested that the β-sheets were the dominant secondary protein structure with the amide I band located at 1656 cm^−1^ (Figure 7c). The β-sheet content in cookie dough could be predictable since this secondary structure was linked to the development of dough elasticity [41]. However, no significant changes were observed in the β-sheet content of all the samples (*p* > 0.05). The peak related to random coils and α-helices was obtained only in a few of the replicates for each sample. Through numerical deconvolution, it was found that the content of random coils was negligible in the dough samples. This was in correlation with a study reported by García-Valle et al. (2021), where it was observed that the random coils were either negligible or absent in the dough made from different cereals [39]. As per the literature, α-helices were considered the primary skeleton for sustaining protein structure since they were more ordered [42]. However, the peaks for α-helices were negligible in the dough samples, suggesting that higher mixing time reduced the orderliness of the protein secondary structures in the dough [43]. Lastly, the content of β-turns followed a similar pattern to that of β-sheets, i.e., no significant variations were observed with increased mixing time. The FTIR spectrum analysis revealed that the preferred secondary structure of the protein in the dough samples was primarily composed of β-turns and β-sheets.

**Figure 8:**
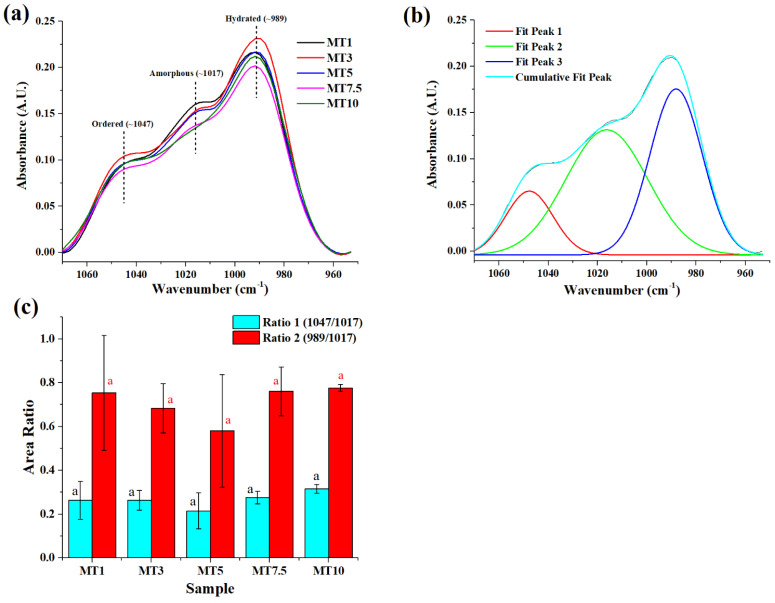
Graphs showing (**a**) starch region in different samples, (**b**) a representative graph of deconvoluted starch region, and (**c**) variation of ratio obtained in the starch region concerning different samples. The alphabets above columns of the same color denote statistically significant (*p* < 0.05) differences.

Moving to shorter wavelengths, a second protein-related peak area at 1463 cm^−1^ was found. The Amide II band is typically found in this region since it is linked to the N-H bending and the C-N and C-C stretching in gluten proteins [44]. A few minor peaks of weak intensity were identified between the wavenumber 1200–1340 cm^−1^. These peaks can be ascribed to the N-H bending and C-N stretching vibrations of the Amide III band [44]. Similarly, the linked C-O and C-C stretching vibrations of polysaccharide molecules, primarily starch, can be associated with the peaks at 1157 and 1103 cm^−1^ [45]. According to García-Valle et al. (2021), the region in the range of 1070–950 cm^−1^ relates to the fingerprint region of the starch and depicts its molecular organization. Therefore, we deconvoluted this region into three Gaussian peaks, i.e., 1047, 1017, and 989 cm^−1^ (Figure 8a,b). Bands at about 1000–995 cm^−1^ are related to hydrated crystalline samples, whereas bands at 1047 and 1017 cm^−1^ are attributed to ordered (e.g., double helices) and amorphous structures, respectively [46]. Rather than directly correlating the peaks to the structures, studies have considered their ratios to quantify the starch structures. The ratio 1047/1017 (Ratio1) and 989/1017 (Ratio2) are evaluated to quantify the short-range order of starch structures and the alignment of helices at the short-order range, respectively [47,48]. For the dough samples, the variation in Ratio1 was similar for all and was in the 0.5–0.8 range. This means that bonded water provides structural order to the starch molecular organization in the dough samples. Ratio2 also followed a similar pattern to that of Ratio1. The last peak, located at around 700 cm^−1^, was attributed to the out-of-plane bending of the hydroxyl groups, which was occasionally associated with free water molecules [49] (Figure 5a).

#### 3.1.5. Texture Analysis of Dough

**Figure 9:**
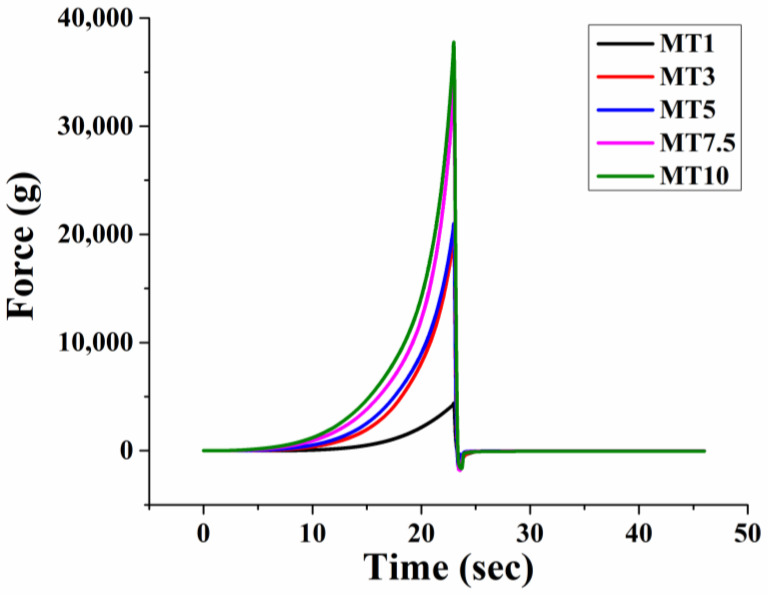
Spreadability profile of cookie dough.

Spreadability is a qualitative, sensory property that may be described as the stress necessary to distribute a material uniformly across a surface. Therefore, the spreadability of dough is a crucial functional attribute since it indicates how simple the dough is to work with and how little effort is required to form sheets. Spreadable food yield points provide manufacturers with a new metric for monitoring and raising production quality standards [50,51]. We used the spreadability rig to measure the ease with which the dough samples could be spread. Firmness, work of shear, stickiness, and work of adhesion were only a few of the derived parameters that were determined after the samples were tested [52]. Figure 9 depicts the dough samples’ spreadability profiles, while Table 2 tabulates the obtained parameters.

##### Firmness

The firmness and stickiness of food items are crucial qualities that influence both their texture in the mouth and their capacity to be processed [53]. The amount of deformation that occurs when applying a particular force may be used to determine how firm or hard the sample is. As observed from the graph, the positive peak force resembles the firmness of the prepared dough samples. MT1 dough was observed to have a significantly lower firmness (~4382 g) than the rest of the samples (*p* < 0.05). This suggests that the water–flour system was not adequately mixed, resulting in less gluten development. Since gluten production is essential for keeping the flour together, a lack of it might result in undesirable cookie properties (such as being more friable). Further, the under-mixing of the dough can lead to a stiff dough after refrigeration, leading to poor gas retention during the baking process. Less air incorporation into the cookies will lead to a dense and firm crumb. A further increase in the mixing time increased the firmness in MT3 and MT5. Such a result is indicative of the fact that higher gluten development has taken place in MT3 and MT5 compared to MT1. However, both samples had similar firmness values (*p* > 0.05). Lastly, MT7.5 and MT10 dough samples showed significantly higher firmness than samples with lower mixing time (*p* < 0.05). As mixing causes the gluten strands to get longer and stronger, excessive mixing can lead to a higher and undesirable gluten content that makes the dough stiff and non-stretchy [26].

##### Work of Shear

The work of shear is intended to describe the amount of work required to spread the dough [54]. The area under the maximum positive peak indicates the work of shear performed on a particular sample. It is to be anticipated that in order to disseminate samples with a higher stiffness concurrently, a greater amount of shear effort will be required. The present study found that the longer we mixed the cookie dough, the more work of shear was needed to spread a sample of the dough. There is often a strong correlation between the work of shear (spreadability) and firmness measures [52]. The findings also demonstrate that the work of shear followed a similar pattern to the dough sample’s firmness (Table 2). In terms of spreadability, we can anticipate that the dough requiring a high amount of shear is less spreadable and vice versa. This indicates that an increase in the mixing time decreases the easiness of spreadability of the cookie dough.

##### Stickiness

The stickiness of dough may be defined as either adhesion of dough to the contact surface or the particles’ cohesiveness [55]. Adhesion is the degree to which a particle is stuck to a surface [56]. Stickiness is the energy needed to remove the working plate from the dough surface after compression. The maximum negative peak represents the stickiness of the sample, and the work of adhesion is calculated as the maximum negative area. To extract the probe from an adhesive sample, more force must be used, thereby increasing the area of the negative region. In the current study, dough stickiness varied between 648 and 2240. The stickiness was the lowest for MT1 and highest for MT7.5. The stickiness values of MT1 and MT7.5 were significantly different (*p* < 0.05). However, the stickiness values of all the other samples were similar to that of MT1 and MT7.5. Wang et al. [57] reported that the differences observed in the dough stickiness might be due to the change in gliadin content in the flour [57]. As observed from FTIR analysis, an increase in the mixing time leads to the release of water molecules from the dough matrix and forms agglomerates. Such water molecules not bound to the dough matrix (especially proteins and fibers) can increase the dough’s stickiness [55]. Lastly, the work of adhesion was similar for all the dough samples. The spreadability study suggests that the MT3 and MT5 dough samples showed better spreadability properties.

#### 3.1.6. Stress Relaxation of Dough

**Figure 10:**
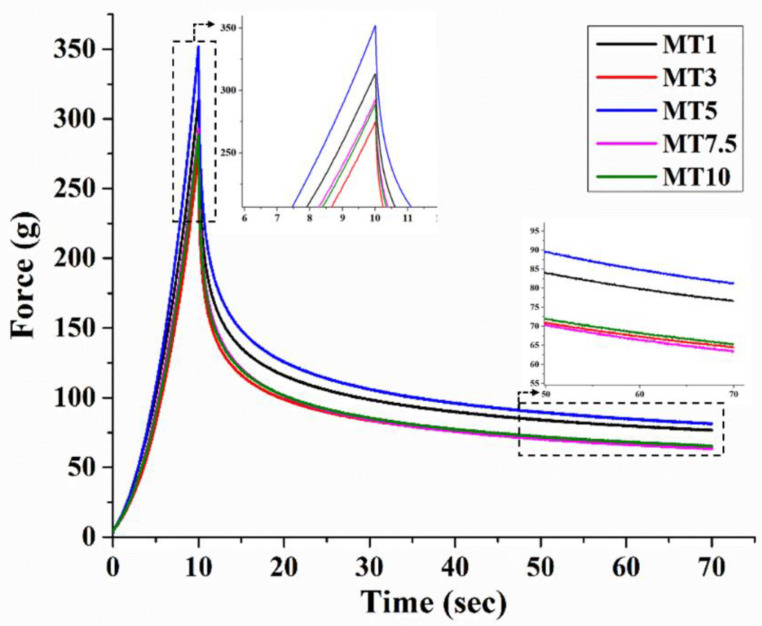
Stress relaxation profile of cookie dough.

Gluten proteins in the dough, when wet, form a three-dimensional network that gives dough its distinctive viscoelastic behavior [58]. Stress relaxation in dough occurs in two phases: the first phase occurs at extremely short relaxation intervals (0.1–0.5 s), while the second phase occurs at slightly longer relaxation times (>10 s) [59]. The variations in the distribution of gluten proteins are accountable for the deformation-induced stress variations in the dough samples. Figure 10 depicts the stress relaxation profile of the dough samples. To learn about the dough samples’ elastic properties, we performed a series of calculations using a macro and compared the results (Table 3).

The maximum force (F_0_ values) under strain revealed the firmness of the dough samples (Table 3). Significant shifts in the F_0_ values of dough samples were seen when mixing time was increased. It was observed that MT1 has an average F_0_ of 313.38 ± 18.56 g. As the mixing time was increased, the F_0_ values decreased significantly in the MT3 dough (*p* < 0.05). A further increase in the mixing time led to a significant rise in the F_0_ value in MT5 (352.12 ± 15.34 g), compared to MT1 and MT3 (*p* < 0.05). As the mixing time reached 7.5 min, a significant reduction was observed in the F_0_ value of MT7.5. The final sample, MT10, had insignificant changes compared to MT7.5. However, the F_0_ values of MT7.5 and MT10 were found to be similar to that of MT1 and MT3. It has been suggested in the literature that if mixing continues for a longer time, the dough becomes softer, less resistant to the mixing process, and loses its ability to retain gas during the resting phase [60]. This process is usually referred to as dough breakdown. Herein, the dough breakdown might have occurred in the MT7.5 and MT10 dough.

When the probe was kept in the same position after reaching a distance of 5 mm, the force values decreased exponentially to a residual force (F_60_). The change in force levels may be traced back to the molecular rearrangement of the dough’s components. A measure of dough’s elasticity is the force that has remained after the test has been halted. Dough composition can influence the force decay profiles. The study showed no variation in the F_60_ values across the dough samples.

The % SR describes the sample’s capacity to absorb the energy associated with the generated strain. A larger % SR is found in materials with a higher degree of fluidity or polymeric structures with easily disruptible networks. Gliadin and glutenin proteins were responsible for the dough’s sticky and elastic properties [61]. Gliadins are monomeric proteins that add to the product’s viscosity, whereas glutenins are polymers that add to the product’s elasticity [62]. In spite of this, moisture levels and cross-linking during baking have an impact on the resulting viscoelasticity [63]. Due to their viscoelastic properties, the force values in doughs reduced swiftly and dramatically over time. Perhaps the dough’s improved malleability can be attributed to the stretching and cross-linking of the gluten network. Each dough sample had a % SR of more than 75%. MT1 and MT3 showed a similar % SR value (*p* > 0.05). MT5 exhibited a significantly higher % SR. value compared to MT1 (*p* < 0.05); however, the value was similar to that of MT3 (*p* > 0.05). MT7.5 showed a significantly higher % SR value than the samples with lower mixing time. MT10 displayed a higher % SR than MT1, but the value was similar to the rest of the samples. However, all things considered, a longer mixing time resulted in a higher % SR. The important step in forming WWF dough is increasing consistency during the mixing process. This phase is usually known as the dough development phase. As the flour particles (agglomerates of the starch granules) hydrate, it forms a viscous slurry where the starch granules get embedded within a gluten protein network. As the mixing time increases, the agitation of the dough causes the starch granules to become loosely attached to the protein fibers. After a critical mixing time is reached, the chemical bonds in the gluten network start to cleave, and a decrease in the dough consistency is observed. Therefore, we can hypothesize that the breakdown of the gluten protein into smaller particles acts as a reinforcing agent in the starch matrix, which increases the % SR of the dough samples [64,65].

#### 3.1.7. Impedance Analysis of Dough

**Figure 11:**
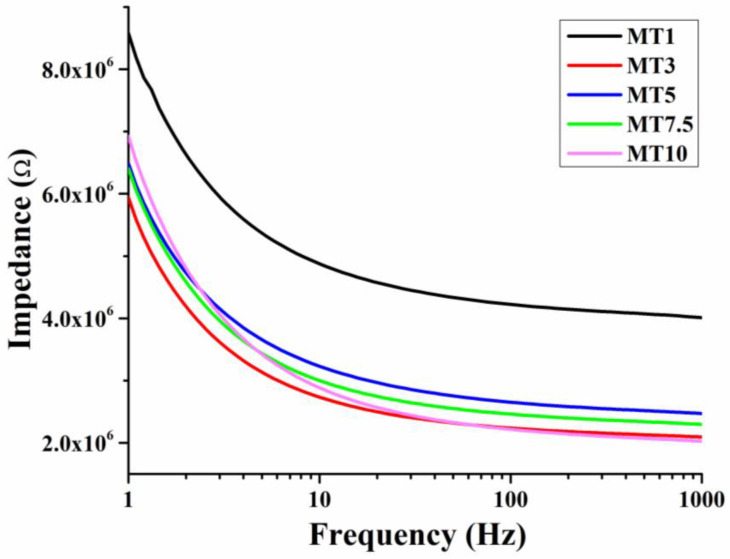
Impedance spectroscopy profile of WWF dough.

The technique of electrical impedance spectroscopy (EIS) has been widely used to study the frequency dependence of electrical characteristics of materials. Numerous EIS applications have been developed to define and screen various food items to evaluate their quality. Moisture is a crucial property in many food products, and EIS may be used to conduct fast, non-destructive measurements of this parameter [66]. Figure 11 represents the impedance profiles of the cookie dough processed with different mixing times. MT1 dough mixing showed a higher impedance than the rest of the samples. Such a higher impedance level can be attributed to the inhomogeneity of the resultant water–flour system [67]. This indicated that cookie dough was under-mixed after a 1 min mixing period. The impedance of MT3 dropped from 1 to 3 min into the mixing process. Impedance went up marginally in MT5. Compared to MT3 and MT5, MT7.5 has a higher impedance. Adding more time to the mixing process increased the impedance at low frequencies. Still, MT10 had an impedance that was on par with MT3 at higher frequencies. Analysis of impedance shows that the water–flour system produced by MT3 is probably relatively consistent. This can be correlated to the MT3 micrograph (Figure 3), which shows the smaller water droplets homogenously distributed throughout compared to other samples. Additionally, WWF’s higher dietary fiber content can lead to a higher water-holding capacity. The amalgamation of dietary fiber and water might have led to the formation of ions. The increase in the ions must have increased the conductivity of MT3 but resulted in a lowering of the impedance [68].

### 3.2. Analysis of Cookies

#### 3.2.1. Visual Appearance of Cookies

As soon as the cookie dough is placed in a preheated oven, baking will begin. During the baking process, the butter in the cookie dough will begin to melt. Rising temperatures during baking speed up the processes (such as the gelatinization of starches and the reticulation of gluten) happening on the inside of the dough. However, at this higher temperature, there is water evaporation. These changes can only happen to a certain extent due to a lack of accessible water molecules. The dough’s moisture turns into steam ~212 oF, which causes the cookie dough to begin to rise. Since baking soda or powder decomposes into carbon dioxide gas, the cookies continue to rise. Hence, the mature cookies have a light and flaky texture because of the small holes caused by these gases.

**Figure 12:**
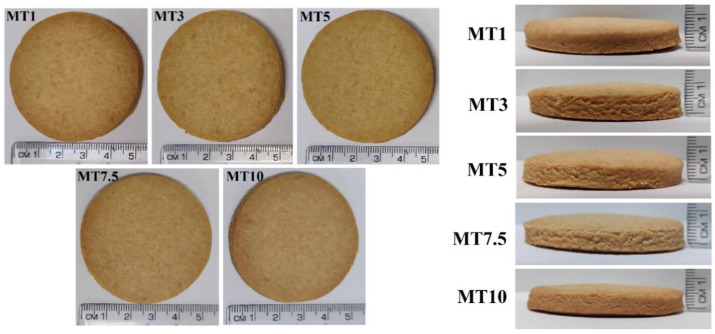
Visual appearance of the cookies.

The baked WWF cookies prepared in this study had a light-brown color (Figure 12), which can be attributed to the crystallization of sucrose during the baking process. Browning of edges was also noticed in baked cookies, indicating that browning begins at the edges and progresses toward the center. Such occurrences have been claimed to indicate baking level, i.e., under-baked, appropriately cooked, and over-baked [69]. The prepared cookies were baked to an optimum level, as appears from the browned edges with a pale center. The visual appearance was uncompromised with the change in the mixing time (Figure 12a). On the surfaces of all cookies, there was an adequate amount of cracking, usually observed in the wheat flour cookies [70]. Figure 12b suggests that there have been textural changes and a rise in the cookies during the baking process.

#### 3.2.2. Surface Topology

**Figure 13:**
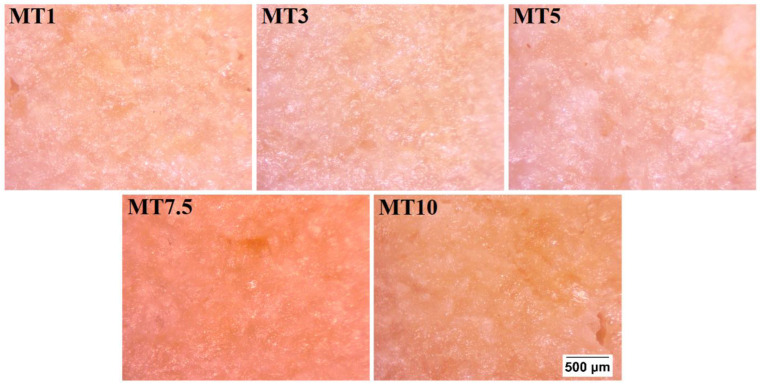
Surface topographs of cookies.

Surface texture is widely recognized as a crucial sensory component of food materials, and it significantly influences customers’ perception and anticipation of a food product. Consumers and researchers are increasingly using surface topology to define food components in terms of their surface and appearance [71]. Figure 13 represents the surface topology of the baked cookies. The surface of the cookies was found to be uneven.

#### 3.2.3. Physical Dimensions

**Figure 14:**
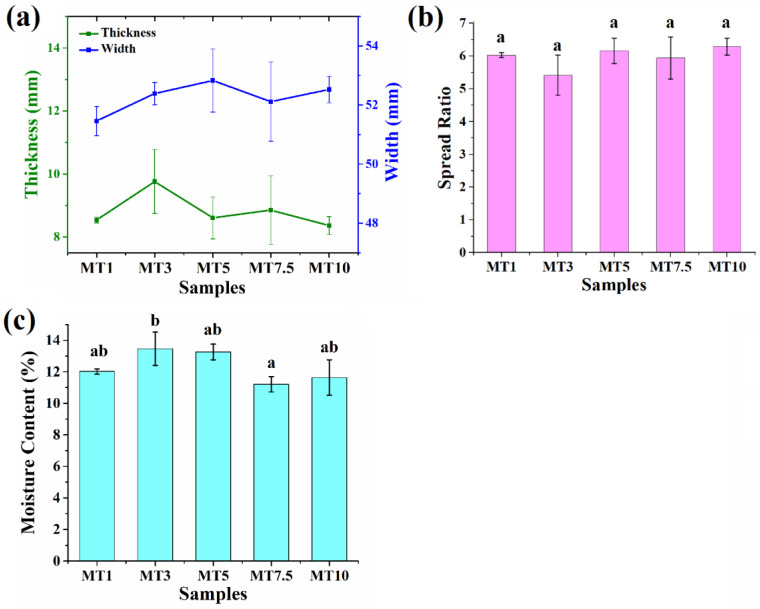
Parameters of cookie samples: (**a**) dimensions of the cookies, (**b**) spread ratio of the cookies, and (**c**) moisture content (%) of the cookies. Error bars represent the standard deviation of three replicates. For each chart, those not sharing the same letter(s) are significantly different by Tukey HSD test.

Cookie dough is turned into a cellular solid with a particular final texture during baking. Leavening dough expansion and gravity flow both contribute to the cookie’s spread. The spread rate and time setting determine the final cookie diameter. Figure 14a depicts the dimensions of the baked cookies, including their width, thickness, and spread ratio. The size and distribution of the cookies changed depending on how long they were mixed. However, the statistical study of cookie samples reveals no statistically significant variation in the values of width and thickness when taking spread into account (Figure 14b). Generally, a greater spread ratio is considered a desirable quality of cookies [72].

#### 3.2.4. Moisture Content

The quantity of water used in cookie preparation significantly impacts the cookie’s texture and its overall attractiveness to consumers. Baked goods’ friability (easiness of breaking apart) is directly proportional to their moisture level [73]. Products having a moisture level of 10% or more are less friable than those with a moisture content of 5% or less. The moisture level of cookies is influenced by variables such as the kind of flour, ingredients used, and processing conditions. Softness and moisture loss are the most noticeable quality changes in baked goods during storage and are indicative of the staleness process. Figure 14c displays the amount of moisture in the cookies. The moisture content of the baked cookies ranged between 11 and 13.5%. However, the moisture content of all the samples was similar except MT3 and MT7.5. MT3 showed significantly higher moisture content than MT7.5. Chauhan et al. [74] reported a similar moisture level as they prepared cookies from whole wheat flour and amaranth flour. Such a high level of moisture content can be attributed to the high fiber content in WWF as compared to other flour-based cookies.

#### 3.2.5. FTIR of Cookies

**Figure 15:**
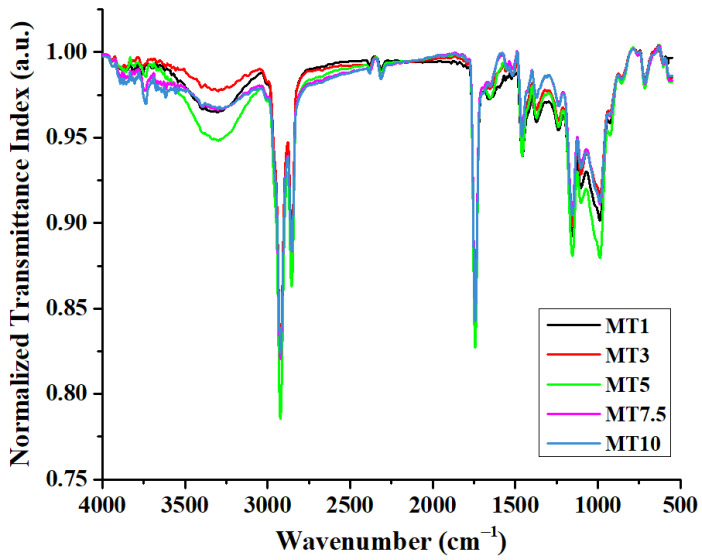
FTIR spectra of cookie samples.

FTIR spectra give structural information and can be used to investigate the interaction of functional groups during food product processing. However, when evaluating the FTIR spectrum data of complex materials such as biscuits, one must use caution since they include several functional groups and their spectra overlap. All the cookie samples showed a broad peak in the region in the range of 3500–3000 cm^−^^1^, which corresponds to the O-H stretching vibrations of the water molecules (Figure 15). Interestingly, the peak for O-H bonding was of the highest intensity in MT5 and lowest in MT3. The notable peaks related to the C-H stretching mode can be readily recognized in all the cookie samples in the wavenumber range of 3000–2800 cm^−^^1^, C=O stretching in the region of 1800–1600 cm^−^^1^ [75], and C-O-C stretching and C-H bending in the region of 1500–650 cm^−^^1^ [76].

#### 3.2.6. Texture Analysis of Cookies

The texture of the cookies contributes much to their overall deliciousness. One of the most important characteristics of a cookie’s texture is its hardness, quantified by the force needed to break it. The composite matrix of protein aggregates, lipids, and sugars found in ungelatinized starch granules is thought to contribute to the hardness of cookies, at least according to published research [77,78]. In terms of creating a composite matrix from cookie dough, variations in total protein concentration were not as noticeable as those in gluten content. However, rather than that, the gluten content was the most notable difference. It is well researched that the gluten network formation increases with an increase in the mixing time; however, over-mixing might negatively affect the gluten network and, eventually, the dough [79]. Refined flours usually have higher gluten content, while in WWF, a higher share of non-gluten protein is present. This makes WWF more convenient for cookies [80]. The presence of high fiber content in WWF can increase the hardness of cookies. Fiber can make cookies more dense and crunchy by absorbing water and increasing the overall structure of the cookie dough. However, it is essential to note that too much fiber can result in dry, crumbly cookies. Due to these reasons, we can expect a higher hardness value for the cookies prepared in the present study.

**Figure 16:**
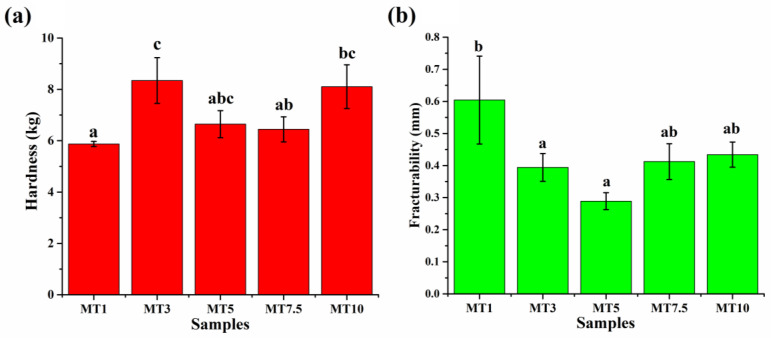
Bar plot of (**a**) hardness and (**b**) fracturability of WWF cookies prepared with different mixing times. Error bars represent the standard deviation of three replicates. Those not sharing the same letter(s) for each chart are significantly different by Tukey HSD test.

Figure 16 is a bar plot depicting the hardness and fracturability of the cookies. The cookie sample that requires the highest force to break will have the highest hardness. MT1 showed a hardness value of ~5872 g, which is the lowest among all the cookie samples. An increase in the mixing time to 3 min resulted in the cookies with hardness values significantly higher than MT1 (8344 g; *p* < 0.05). Such a difference could be expected due to the lower O-H bonding in MT3 than in MT1. This could have easily prevented water molecules from entering the MT3 cookies. As the mixing time increased to 5 min, there was a remarkable decrement in the hardness of MT5 cookies. Additionally, in the moisture analysis, the removal of bound water in MT3 was inhomogeneous compared to MT5, which showed a lower deviation. Similar observations were also found in hardness, where we observed higher deviation in the case of MT3 compared to MT5. Further, the statistical evaluation showed that MT7.5 had a hardness value similar to that of MT5 (*p* > 0.05). In MT10, the hardness value again increased, which was more similar to MT3. Overall, the hardness value of the cookies varied with an increase in the mixing time [81,82].

The amount (in millimeters) that a cookie sample deforms until it breaks when it is compressed is called its “fracturability” or “brittleness” [23]. MT1 showed the highest fracturability value among all the samples. An increase in the mixing time resulted in a decrease in the fracturability value. There were no significant differences in fracturability values of MT3 and MT5 cookies. Interestingly the fracturability value of the MT5 cookie was more reproducible than the rest of the samples. However, the values were significantly lower from MT1 (*p* < 0.05). Lastly, MT7.5 and MT10 showed statistically similar fracturability values regardless of different mixing times (Figure 16b). From the texture analysis, we concluded that MT of 1 min results in significantly higher fracturability and MT5 with the lowest fracturability.

## 4. Conclusions

This research sought to determine the relationship between the mixing time of WWF-based cookie dough and the cookie’s properties. In this study, microscopy, texture (spreadability and stress relaxation), moisture content, and impedance analysis were used to assess cookie dough qualities over a range of mixing times. No major changes in the dough’s moisture content were observed because the same ingredients and their amount were used for each sample. The microscopic analysis revealed major changes in the distribution of water molecules in all the dough samples. Interestingly, MT3 showed a better distribution of the ingredients throughout the sample matrix. The segmentation analysis of the dough micrographs suggested that higher mixing time resulted in the formation of water agglomeration. The numerical deconvolution of the water region (3800–3000 cm^−^^1^) revealed that the bound water molecules started to isolate from the dough matrix with an increase in the mixing time. The analysis of the amide I region (1700–1600 cm^−^^1^) suggested that β-turns and β-sheets were the dominating protein secondary structures in the dough matrix. Starch region analysis suggested that most of the starch in the dough matrix was in the hydrated form. The dough samples were analyzed to determine their textures, and the MT3 dough was found to have the best balance of firmness and stickiness. It was also found via the impedance study that MT3 dough had the lowest impedance.

We also evaluated the spread factor, surface topology, moisture content, and textural characteristics of the baked cookies. The yellow coloration of the cooked cookies was traced back to the sucrose crystallizing in the oven. There was no noticeable difference in appearance despite the alteration in the mixing time. All of the cookies had significant surface cracking, usually associated with the wheat flour cookies, which contributed to their overall impression of the unevenness of the cookie surface. There was no statistically significant variation in the values of the width and thickness of the cookies. Approximately 11–13.5% of the total weight of the cookies was moisture. However, the moisture content of MT3 was higher than the rest of the samples. The FTIR spectrum of cookie samples showed that the peak related to the hydrogen bonding was of high intensity in MT5 than in the rest of the cookie samples. The texture analysis of cookies showed that the hardness of the cookies varied with a change in the mixing time. MT5 cookies had the lowest fracturability value. Overall, it was observed that the MT3 dough and MT5 cookies were better than the rest of the samples. Hence, it can be concluded that a creaming time of 5 min and a mixing time of 5 min was best suited for whole wheat flour-based cookies.

## Figures and Tables

**Table 1:** Composition of cookie and parameters of baking.

Ingredients	Quantity (g)	Formulations	Creaming Time (min)	Mixing Time (min)	Baking Time (min); Temperature (°C)
Whole wheat flour	100	MT1	5	1	25; 180
Butter	40	MT3	5	3	25; 180
Sugar	40	MT5	5	5	25; 180
Salt	1	MT7.5	5	7.5	25; 180
Baking powder	3	MT10	5	10	25; 180
Water	25				

**Table 2:** Parameters obtained from the spreadability test of cookie dough. For each column, different letter(s) indicates significantly different values by the Tukey HSD test. The values were represented as Avg ± Stdev.

Sample	F1 (g) Firmness	Positive Area (g s) Work of Shear	F2 (g) Stickiness	Negative Area (g s) Work of Adhesion
MT1	4382.02 ± 467.77 ^a^	16,457.18 ± 1305.08 ^a^	−648.30 ± 59.20 ^b^	−807.46 ± 213.34 ^a^
MT3	21,030.84 ± 1899.85 ^b^	65,836.97 ± 4269.83 ^b^	−1140.15 ± 59.05 ^ab^	−1537.45 ± 990.58 ^a^
MT5	22,188.50 ± 445.71 ^b^	74,909.47 ± 2922.05 ^b^	−1781.81 ± 279.29 ^ab^	−617.85 ± 41.05 ^a^
MT7.5	35,313.60 ± 5545.35 ^c^	109,079.59 ± 15,739.49 ^c^	−2239.42 ± 532.64 ^a^	−860.64 ± 277.00 ^a^
MT10	39,988.39 ± 1160.97 ^c^	128,045.37 ± 3019.22 ^c^	−1779.37 ± 999.53 ^ab^	−1318.85 ± 911.76 ^a^

**Table 3:** Stress relaxation parameters. For each column, different letter(s) indicates significantly different values by the Tukey HSD test.

Sample	Stress Relaxation Parameters (Average ± Standard Deviation)		
	F_0_ (g)	F_60_ (g)	%SR
MT1	313.38 ± 18.56 ^b^	76.52 ± 3.87 ^b^	75.57 ± 0.59 ^a^
MT3	275.07 ± 9.47 ^a^	64.36 ± 1.69 ^a^	76.60 ± 0.21 ^ab^
MT5	352.12 ± 15.34 ^c^	81.25 ± 2.39 ^b^	76.92 ± 0.33 ^b^
MT7.5	293.21 ± 14.36 ^ab^	63.21 ± 3.96 ^a^	78.45 ± 0.51 ^c^
MT10	289.05 ± 8.63 ^ab^	65.37 ± 2.43 ^a^	77.39 ± 0.30 ^bc^

## Data Availability

Not applicable.
